# Molecular detection of the zoonotic trematode *Centrocestus formosanus* (Nishigori, 1924) (Opisthorchiida, Heterophyidae) in Central Europe

**DOI:** 10.1007/s11259-024-10636-1

**Published:** 2025-01-08

**Authors:** Ľubomír Šmiga, Júlia Šmigová, Federica Berrilli, Ingrid Papajová, Peter Lazár, Isabel Guadano-Procesi

**Affiliations:** 1 Department of Breeding and Diseases of Game, Fish and Bees, Ecology and Cynology, University of Veterinary Medicine and Pharmacy in Košice, Komenského 73, Košice, 041 81 Slovakia; 2 Institute of Parasitology, Slovak Academy of Sciences, Hlinkova 3, Košice, 040 01 Slovakia; 3 Department of Clinical Sciences and Translational Medicine, Faculty of Medicine, University of Rome “Tor Vergata”, Via Montpellier 1, Rome, 001 33 Italy

**Keywords:** Gill parasite, Heterophyidae, Ornamental fish disease, Ornamental fish trade, Platyfish, Trematodiasis, *Xiphophorus maculatus*

## Abstract

In our study, fancy southern platyfish *Xiphophorus maculatus* (Cyprinodontiformes, Poeciliidae) were examined due to breathing disorders and mortality. Fish came from Vietnam farm and were redistributed by international wholesaler. In fish, loss of appetite and gasping near the water surface was observed. Gill tissue showed small white spots, caused by metacercariae and areas of clearing surrounding the cysts. Primary branchial filaments were hyperplastic, necrotic, significantly deformed and shortened. Samples were fixed in 98% alcohol for molecular analyses. The identity of *C. formosanus* (Opisthorchiida: Heterophyidae) was confirmed by molecular methods (GenBank accession number OP808358). In this study, we present the first report of *C. formosanus* in Central Europe. Uncontrolled transport of fish can lead to health and economic concerns, including the transmission of zoonotic pathogens to non-native ecosystems.

## Introduction

Asian countries (as Singapore, Indonesia, Thailand, Sri Lanka, Japan, Vietnam and China) are one of the most important exporters of ornamental fish to European Union (The Ornamental Aquatic Trade Association, EU Ornamental Fish Import & Export Statistics 2016). There is a big potential risk of worldwide pathogen transmission through live aquatic animals, which are not adequately controlled in quarantine facilities (Scholz and Salgado-Maldonado, 2000; Evans and Lester, 2001; Bassleer, 2009).

The parasitic Platyhelminthes *Centrocestus formosanus* (Nishigori, 1924) (Opisthorchiida: Heterophyidae) is a digenean trematode that requires three host species to complete its life cycle (Chen, 1942). The first intermediate hosts are aquatic mollusks (Thiaridae), the second intermediate hosts are fish, frogs (genus *Rana*) and definitive hosts can be mainly piscivorous birds (as *Nycticoras* or *Butorides* were reported, almost any fish eating bird should be considered as a possible host) or mammals (rats, cats, foxes and dogs), including humans (Mitchell and Goodwin, 2004; Chai et al. 2009; Chai et al. 2013). Human infections were recorded in Lao People’s Democratic Republic (Chai et al. 2013) and Vietnam (De and Le, 2011). In other cases originating from Japan, China, or Taiwan, just a probable infection caused by *C. formosanus* was recorded, without a more precise determination of the parasite (Chai et al. 2013).

*Centrocestus formosanus* is considered a generalist parasitic species with low intraspecific variability among different hosts (Scholz and Salgado-Maldonado, 2000). Aquarium fish species from various families have already been reported as naturally or experimentally infected by this parasite (Yousif et al. 2016; Morales-Serna et al. 2019). This digenean has been detected in approximately 24 egg-laying fish species from seven orders (Table 1) and in 11 livebearer species from order Cyprinodontiformes (Table 2), which came from aquaculture or where detected in aquaculture.

**Table 1:** Occurence of trematode *Centrocestus formosanus *in egg lying ornamental fish

Order	Species	Country	References
Cypriniformes	*Cyprinus carpio *var. *koi*	Mexico, Thailand	[1, 2]
	*Carassius *spp.	Mexico	[1]
	*Carassius auratus*	Iran, Thailand, Turkey	[2, 3, 4, 5, 6]
	*Danio rerio*	Italy, Mexico, Thailand	[1, 2, 7, 8]
	*Puntius brevis*	Laos	[9]
	*Puntigrus tetrazona*	Thailand	[2]
	*Labeo erythrurus*	Iran	[5]
Cichliformes	*Pterophyllum scalare*	Turkey	[4]
	*Nimbochromis venustus*	Mexico	[1, 10]
	*Amatitlania nigrofasciata*	Mexico	
	*Vieja fenestrata*		[10]
	*Cichlasoma geddei*		
	*Thorichthys helleri*		
	*Thorichthys pasionis*		
	*Parachromis managuensis*		
	*Trichromis salvini*		
	*Australoheros facetus*	Brazil	[11]
Anabantiformes	*Trichogaster lalius*	Mexico	[1]
	*Trichopodus trichopterus*		
Osteoglossiformes	*Osteoglossum bicirrhosum*	Iran	[5]
Siluriformes	*Hypostomus plecostomus*	Mexico	[1]
Gobiiformes	*Dormitator latifrons*	Mexico	[10]
	*Gobiomorus dormitor*		
Cyprinodontiformes	*Aplocheilus panchax*	Thailand	[12]

Legend: 1. Ortega et al. 2009; 2. Wanlop et al. 2017; 3. Jaruboonyakorn et al. 2022; 4. Yildiz, 2005; 5. Mood et al. 2010; 6. Taner and Yıldız, 2018; 7. Iaria et l. 2019; 8. Pace et al. 2020; 9. Han et al. 2008; 10. Scholz and Salgado-Maldonado, 2000; 11. Pinto and De Melo, 2010; 12. Madhavi and Rukmini, 1991

*C. formosanus* originated from Asia, described in Taiwan and was later widely transmitted to different Asian countries and worldwide (Chai et al. 2013; Pace et al. 2020), which points to its distinctive invasive character. Both the parasite and snail are now firmly established in the upper and middle reaches of the Comal River, Texas, where the parasite has negatively impacted the local fish fauna (Mitchell et al. 2000). In Mexico 70 species of freshwater fishes (3 species of goodeids and 20 poeciliids) are recorded to host *C. formosanus* in natural ecosystems (Salgado-Maldonado and Rubio-Godoy, 2014).

**Table 2:** Occurrence of trematode *Centrocestus formosanus* in livebearer fish except halfbeaks

Family	Genus	Species	Country	References
Goodeidae	*Ilyodon*	*Ilyodon whitei*	Mexico	[1]
Poeciliidae	*Poecilia*	*P. reticulata*	Australia, Brazil, Mexico, Turkey	[1, 2, 3, 4, 5, 6]
		*P. vivipara*	Brazil	[7]
		*P. velifera*	Mexico	[8]
		*P. latipinna*	Mexico, Thailand	[1, 9]
		*P. sphenops*	Mexico	[1, 5]
	*Gambusia*	*G. affinis*	Egypt	[10]
	*Pseudoxiphophorus*	*P. bimaculatus*	Mexico	[1]
	*Xiphophorus*	*X. maculatus*	Australia, Brazil, Mexico, Singapur-Denmark, Taiwan, Turkey	[1, 2, 5, 6, 7, 11, 12]
		*X. hellerii*	Mexico,Turkey	[1, 5, 13]
		*X. birchmani*	Mexico	[14]
		*Xiphophorus* sp.	Brazil	[4]
Zenarchopteridae	*Dermogenys*	*D. pusilla*	Thailand	[15]

Legend: 1. Scholz and Salgado-Maldonado, 2000; 2. Evans and Lester, 2001; 3. Pinto and De Melo, 2010; 4. Ciccheto et al. 2021; 5. Ortega et al. 2009; 6. Yildiz 2005; 7. Simões et al. 2006; 8. Morlaes-Serna et al. 2019; 9. Wanlop et al. 2017; 10. Yousif et al. 2016; 11. Leibowitz et al. 2019; 12. Mehrdana et al. 2014; 13. Taner and Yıldız, 2018; 14. Bautista-Hernández et al. 2019; 15. Patarwut et al. 2020

In fish came from ornamental fish trade in Europe it was detected in Turkey (Yildiz, 2005; Taner and Yildiz, 2018), in Denmark (Mehrdana et al. 2014), Italy (Iaria et al. 2019; Pace et al. 2020) and Croatia (Gjurčević et al. 2007). The aim of our study was to identify trematode metacercariae and to obtain parasitological parameters associated with this pathogen, which was found in our fish.

## Materials and methods

In 2022, a total of 12 specimens (in equal gender ratio) of fancy southern platyfish *Xiphophorus maculatus* (Günther, 1866) (Cyprinodontiformes, Poeciliidae) were examined. Species determination was based on Fishbase (n.d.) and the author’s experience as a livebearer expert. The fish were of marigold base colour and with natural or highfin shape of the dorsal fin. Fish were imported from a fish farm in Vietnam by international wholesaler with corporate branch in Slovakia for distribution to local pet shops or private aquarists. Examined fish were obtained directly from a supplier by one of the authors (Ľ. Š.) of this article for breeding and subsequent showing of fish at exterior competitions.

In all fish, complete pathological necropsy focused on parasitological examination was undertaken. Details of anamnestic data were recorded into ichthyopathological protocols (included name of fish species and variety, sex, source of samples, standard length, weight before and after necropsy, anamnestic data, results after necropsy, possibly proposed treatment). Metacercariae were found during the initial processing of gill tissue; consequently, parasites were counted. Length and width of metacercarial cysts were measured from live preparates from wet mounts and data are presented in micrometers, the mean followed by the range in parenthesis. The parasites with small piece of gill tissue around were washed with physiological solution and fixed in 98% alcohol for molecular analyses. The intensity of infection (i.i.), the mean intensity of infection (m.i.) as well as the prevalence (P) for each gill leaf (four on either side of the gill cavity) in each examined specimen was recorded. The total parasitic load values for the group were calculated from the partial values of specimens.

### Molecular methods

The identity of the observed metacercariae was ascertained using DNA sequence analysis. Gills with metacercariae were collected from fresh fish during necroscopy and genomic DNA extraction was performed from approximately 50 mg of gills tissue.

Genomic DNA was extracted from gills with metacercariae using the QIAamp DNA Mini Kit (Qiagen, Germany) according to the manufacturer’s instructions for extracting DNA from tissues. Eluted DNA was stored at − 20 °C before further processing.

Amplification of a partial sequence of the internal transcribed spacer 2 (ITS2) gene was using the following primers: forward 3S (5’-GGTACCGGTGGATCACTCGGCTCGTG-3’) and reverse BD2 (5’-TATGCTTAAATTCAGCGGGT-3’) according to work of Wanlop et al. 2017. The resulting fragment was 402 bp in length.

For PCR to amplify the ITS2 fragment was used total volume of 25 µl consisted of 5 µl of extracted parasitic DNA, 1× DreamTaq Green Buffer, 1 mM MgCl_2_, 200 µM of each dNTP, 0.5 µl of 25 pmol of each primer 2.5 U of Taq DNA Polymerase (Qiagen, Germany).

PCR was performed as follows: initial denaturation was performed at 95 °C for 5 min, followed by 35 cycles of denaturation at 94 °C for 1 min, annealing at 55 °C for 1 min and elongation at 72 °C. The final extension was performed at 72 °C for 7 min in the MyCycler™ Thermal Cycler System (Bio-Rad Laboratories, Berkeley, CA, USA). Amplicons were separated on a 1.5% agarose gel stained with Goodview Nucleic Acid Stain (SBS Genetech Co., China) and TAE buffer (40 mM Tris, pH 7.8, 20 mM acetic acid, 2 mM EDTA). Positive PCR products were purified using the ISOLATE II PCR and Gel Kit (Bioline, UK) and sequenced in an automated ABI Prism 3700 DNA sequencer at the University of Veterinary Medicine and Pharmacy, Košice, Slovakia for both strands using the same set of primers as in the respective PCR tests. The obtained DNA sequences were compared with the reference sequences in the GenBank database by the nucleotide BLASTn program (https://blast.ncbi.nlm.nih.gov) and were grouped by similarity and aligned using MEGA 11 (Tamura et al. 2021). The Kimura 2-parameter model was selected as the best fit model for the analyzed data. All positions containing gaps and missing data were removed. *Haplorchis taichui* was selected as the outgroup. Bootstrap analysis was performed with 1,000 replicates to test the robustness of the phylogeny.

The genetic material of the parasites is deposited at the Department of Breeding and Diseases of Game, Fish and Bees, Ecology and Cynology, University of Veterinary Medicine and Pharmacy in Košice, Slovakia.

## Results

**Fig. 1:**
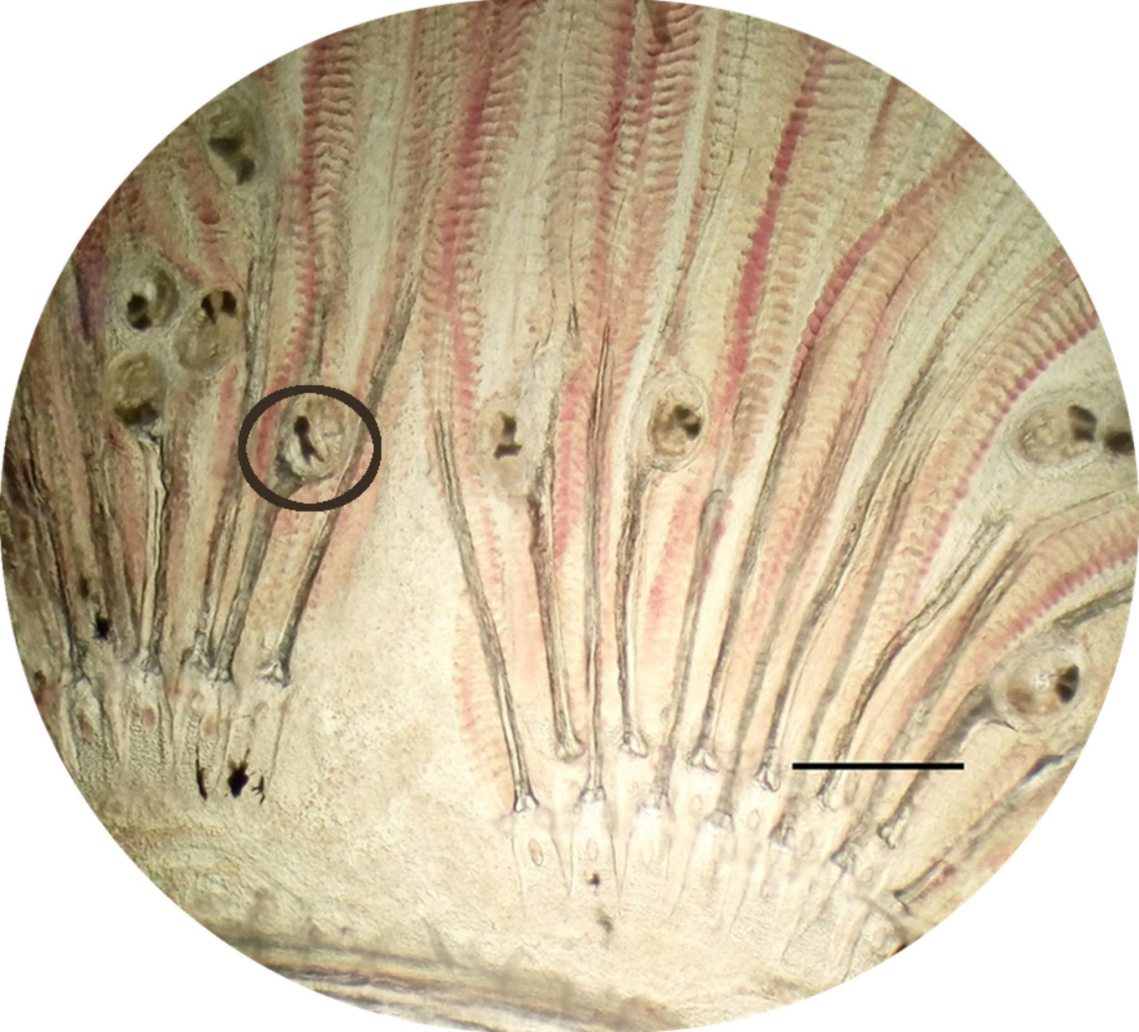
Metacercariae of *Centrocestus formosanus* localization in primary branchial filaments, scale bar 300 μm

Necropsies were performed due to breathing disorders and mortality in the infected platyfish group. In fish, loss of appetite and gasping near the water surface was observed. During the examination, hyperemic and swollen gill filaments with excessive mucous accumulation were observed, especially in the most infected platies. Gill tissue showed small white spots, caused by metacercariae and areas of clearing surrounding the cysts. Parasites were found in primary branchial filaments, especially near the gill cartilage, predominantly in the first third of their length from the gill arch (Fig. 1). Primary lamellae were hyperplastic, necrotic and significantly deformed and shortened. No other parasites were found on/in gills, fins, skin or in body cavities and organs. The ellipsoidal metacercarial cysts were 169 ± 17 μm in length and 135 ± 8 μm in width (*n* = 12). Excysted metacercariae had the excretory vesicles in X-shaped containing dark excretory granules.

The identity of *C. formosanus* was confirmed by molecular methods. At the ITS2 locus, our *C. formosanus* isolate collected herein (OP808358) was 100% identical to sequences of *C. formosanus* from Longfin mojarra (Gerreidae, *Pentaprion longimanus*) in Thailand and from platy (Poeciliidae, *Xiphophorus maculatus*) imported from Singapore to Denmark, archived in GenBank (KX430150, KF658456).

The prevalence of the metacercarial cysts of *C. formosanus* in our fish group was 100% (12/12). The mean intensity of infection was 18.3, with a range of 1–33 parasites per fish. There were no significant differences between right and left gills – on the left gill side was m.i. 9 and i.i. 1–24, on the right side m.i. was 9.3 and i.i. in the range of 3–23. Similarly, no significant differences were observed between sexes; however, males exhibited slightly higher parasitic load values (m.i. 19.3, i.i. 4–33) compared to females (m.i. 17.3, i.i. 1–32). The highest intensities of infection, in the range of 26–33, were observed in dead fish (three specimens).

**Fig. 2:**
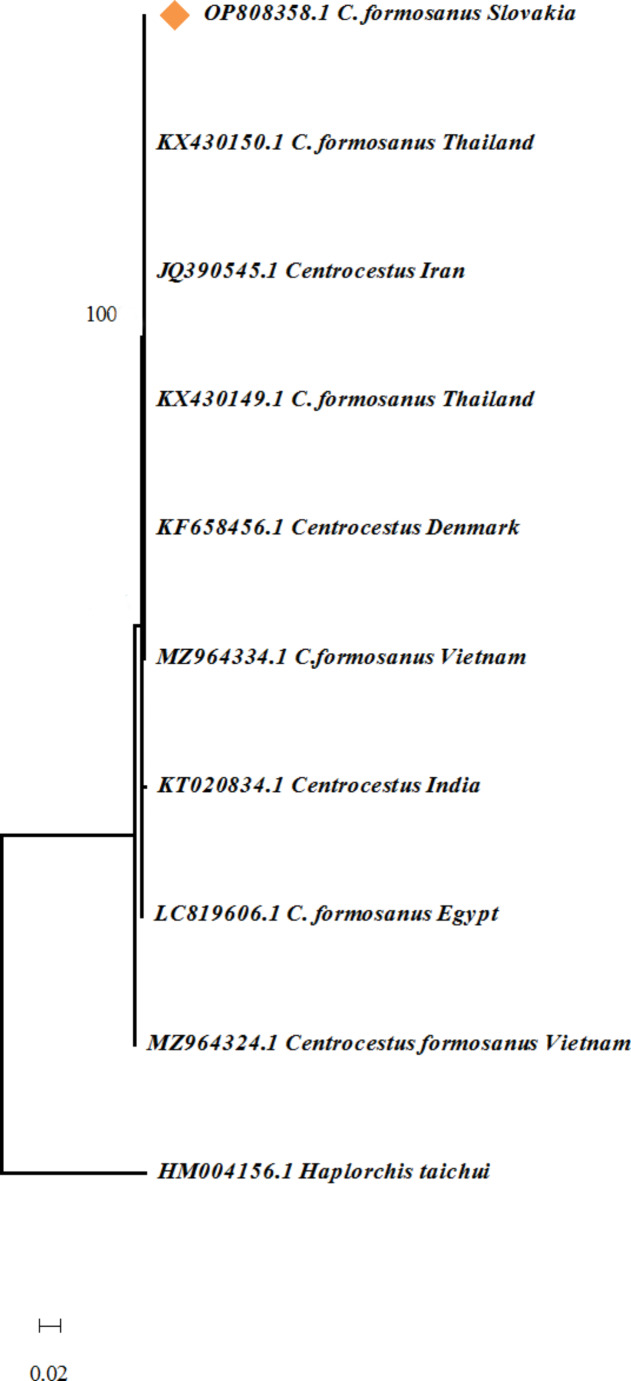
Phylogram generated from ITS sequences by Neighbor-Joining based on Kimura’s 2-parameter method. Tree showing the relationships between the studied isolate of *X. maculatus C. formosanus* from Slovakia (C. formosanus Slovakia) and related sequences retrieved from GenBank. Scale refers to a phylogenetic distance of 0.02 nucleotide substitutions per site. Number next to branches indicate bootstrap values

To illustrate the genetic relationships between our isolates and reference isolates, a phylogram was constructed using the Neighbor-Joining (NJ) method (Fig. 2).

## Discussion

In this study, we present the first report of *C. formosanus* in Central Europe. Similar to findings in other European countries (Gjurčević et al. 2007; Mehrdana et al. 2014; Pace et al. 2020), the parasites were transmitted via the ornamental fish trade and came predominantly from Asian fish farms.

The examination of our fish was initiated by clinical signs as well as fish mortality. Encysted metacercariae were found in the primary lamellae, leading to lamellar deformation. The gill lesions caused by parasite negatively affecting fish welfare, reducing production in fish farms, and threatening biodiversity (Scholz and Salgado-Maldonado, 2000). In our study metacercariae were localized predominantly in the first third of their length from the gill arch. Mehrdana et al. (2014), found cysts were present along the entire length of the primary gill filaments of the same host species. Chen (1942) also detected a small number of metacercariae of this parasite in muscle tissue under the scales of heavily-infected fish. Primodiagnosis was based on characteristic features of *C. formosanus*, such as the very dark X - shaped excretory vesicle occupying the majority of the caudal body portion, as stated by Evans and Lester (2001). Metacercariae were processed through molecular analysis to confirm the identity. Despite a high prevalence and diverse parasitofauna in platyfish (Bassleer 2009) in this study no other parasitic species were found.

In previous studies, the *C. formosanus* was described in one goodeid species and in eleven poeciliids in aquaculture. The parasite was detected in southern platy (*X. maculatus*), our host species, in studies from different parts of the world (Scholz and Salgado-Maldonado, 2000; Evans and Lester, 2001; Yildiz, 2005; Ortega et al. 2009; Mehrdana et al. 2014; Liao et al. 2017; Leibowitz et al. 2019). Several different case reports in the same host of this pathogen may be related to the fact that fancy platyfish are one of the most frequently bred and imported fish species, due to their popularity in the aquarium trade. Despite examining numerous fish from direct Asian imports, *C. formosanus* has not been observed in platyfish or other livebearers such as guppies (*P. reticulata*) or swordtails (*X. hellerii*) in our past studies. This suggests that the parasite’s presence may be related to specific zoohygiene practices and breeding technologies at particular farms (Scholz and Salgado-Maldonado, 2000). A report on the successful establishment of *C. fomosanus* and its first intermediate host, a snail (*Melanoides tuberculata*), was presented by Salgado-Maldonado and Rubio-Godoy, 2014, where natural infection was detected in a huge diversity of new hosts (70 species), also in fish of the genus *Xiphophorus*, specifically in the species *X. hellerii* and *X. variatus*.

A 100% prevalence of *C. formosanus* infection in platyfish was observed in the study reported by Evans and Lester (2001), where guppies and platies were also examined after their import to Australia. In ornamental fish imported from Singapore farm to Turkey for commercial suppliers, the highest prevalence of centrocestiasis was recorded in platies (*P* = 50%), compared to a 20% prevalence in other examined poeciliids as guppies (Yildiz, 2005). High total prevalence from a high number of examined fish (*P* = 40.3%, 278 positive specimens/690 analyzed specimens) was also detected by Ortega et al. (2009) in Mexican farms. In this case, screening was performed on 23 farms and 12 of them were positive for this disease.

The intensity of infection in our study (range 1–33) was lower compared to study from Denmark, where intensity ranged from 195 to 740 parasites per fish (Mehrdana et al. 2014). The mean intensity of infection observed by Mehrdana et al. (2014) (454.5) was also much higher compared with our observations (mean of 18.3 parasites per fish). A slightly lower mean intensity of infection (14.3 parasites per fish) was observed in the bigger *Xiphophorus* species *X. hellerii* (Taner and Yildiz, 2018). The mortality rate was approaching 95% in Brazilian farm-raised platies observed by Leibowitz et al. (2019). In our study, we observed mortality of highly infected specimens, where the intensity of infection was in the range of 26–33. The gill parasites, especially in high intensities, can cause mass mortalities, which leads to serious economic losses through infestation of ornamental fishes. Especialy in aquaculture conditions (such as high stocking density, handling during the cleaning and transport, small water level, poor water quality, etc.) can lead to an increase of intensity of parasitic infection and consequently high mortalities among farmed fish. Although exporting conditions in the aquaculture of ornamental fish (characteristic with closed aquaculture systems) allow parasites with a direct life cycle to attain high prevalence of infection, parasites with an indirect life cycle as digenean trematodes or heteroxenous nematodes are also able to infect fish (Evans and Lester, 2001; Kim et al. 2002). Uncontrolled transport of fish can lead to health and economic concerns, including the transmission of pathogens from imported ornamental fish to European farmed aquarium fish, food fish populations, or wild populations of native fish species (Scholz and Salgado-Maldonado, 2000). New parasitic species can have also negative effects on the natural environments of importing countries and in the case of zoonotic species also on public health (Kim et al. 2002; Chai et al. 2013).

## Data Availability

No datasets were generated or analysed during the current study.
